# Observations on Carbapenem Resistance by Minimum Inhibitory Concentration in Nosocomial Isolates of *Acinetobacter* species: An Experience at a Tertiary Care Hospital in North India

**Published:** 2008-06

**Authors:** A. Gaur, A. Garg, P. Prakash, S. Anupurba, T.M. Mohapatra

**Affiliations:** Department of Microbiology, Institute of Medical Science, Banaras Hindu University, Varanasi (UP) 221 005, India

**Keywords:** *Acinetobacter*, *Acinetobacter baumannii*, Antibiotic resistance, Carbapenem, Cross-infections, Drug resistance, Microbial, Meropenem, India

## Abstract

*Acinetobacter* species are emerging as an important nosocomial pathogen. Multidrug-resistant *Acinetobacter* spp. has limited the option for effective treatment. Although carbapenems are effective for the treatment of such infections, resistance to this drug has recently been reported. This study was undertaken to assess resistance to carbapenem in clinical isolates of *Acinetobacter* spp. from hospitalized patients by both disc-diffusion and minimum inhibitory concentration (MIC) methods. All clinical samples from suspected cases of nosocomial infections were processed, and 265 isolates were identified as *Acinetobacter* species. These isolates were tested for antibiotic resistance by the disc-diffusion method with 14 antimicrobials, including meropenem and imipenem. Thereafter, all *Acinetobacter* species were subjected to MIC for meropenem. More than 80% resistance to second- and third-generation cephalosporins, aminoglycosides, and quinolones was recorded. Thirty percent of the strains were resistant to cefoperazone/sulbactam. Resistance to meropenem was observed in 6.4% of *Acinetobacter* spp. while 8.3% of the isolates showed intermediate resistance detected by MIC. All carbapenem-resistant/intermediate strains were also resistant to other (>10) antibiotics tested by the disc-diffusion method. The rising trend of resistance to carbapenem poses an alarming threat to the treatment for such infections. Regular monitoring, judicious prescription, and early detection of resistance to carbapenem are necessary to check further dissemination of drug resistance in *Acinetobacter* spp.

## INTRODUCTION

Although genus *Acinetobacter* was originally identified in the early 20th century, it was recognized as a ubiquitous pathogen only in the last decade (1). *Acinetobacter baumannii,* a member of the *Acinetobacter calcoaceticus—A. baumannii* complex, makes up to 73% of all *Acinetobacter* spp. and is the most commonly-involved pathogen in clinical infections (2). During the last decade, hospital-acquired infections involving multidrug-resistant *A.* *baumannii* isolates have been reported, often in association with contamination of hospital equipment or cross-contamination by colonized hands of personnel attending patients (1). Initial concern about multidrug-resistant and carbapenem-resistant *Acinetobacter baumannii* (CRAB)-associated infections began when the first hospitalwide outbreak occurred in New York city in 1991 (3). Since then, reports of CRAB have been accumulating from other parts of the world (4), including India (5). Currently, the spread in hospital populations of resistant microorganisms is of great concern worldwide, suggesting that we may be approaching the post-antimicrobial era (6). This study was undertaken to assess resistance to carbapenem in clinical isolates of *Acinetobacter* spp. from hospitalized patients by both disc-diffusion and minimum inhibitory concentration (MIC) methods.

## MATERIALS AND METHODS

### Duration and place of study

A three-year study (2003–2006) was conducted to determine the susceptibility of nosocomial isolates of *Acinetobacter* spp. to different antimicrobials, including imipenem and meropenem. Various specimens were collected from patients admitted to different wards and intensive care unit of S.S. Hospital, Banaras Hindu University, Varanasi, India.

### Identification of *Acinetobacter* spp.

Isolation of A*cinetobacter* spp. was done. Briefly, all clinical specimens were initially processed to separate the oxidase-negative, non-fermenters from other gram-negative bacilli. Thereafter, identification was done to confirm *Acinetobacter* spp. by standard protocol (7).

#### In vitro susceptibility

Susceptibility to various antimicrobial agents was determined by the disc-diffusion method and MIC by the agar dilution method following the guidelines of Clinical and Laboratory Standards Institute (CLSI). Antimicrobial susceptibility testing was performed on Mueller Hinton agar by the disc-diffusion method for the following antimicrobial agents (Hi-Media, Mumbai, India) with their concentration given in parentheses: cefotaxime (30 μg), ceftazidime (30 μg), cefoperazone (75 μg), ciprofloxacin (05 μg), norfloxacin (10 μg), amikacin (30 μg), gentamicin (10 μg), tobramycin (10 μg), netilmicin (30 μg), piperacillin (100 μg), carbenicillin (100 μg), cefoperazone/sulbactam (75 μg/30 μg), meropenem (10 μg), and imipenem (10 μg) by the Kirby-Bauer method. Further *in vitro* susceptibility was determined for meropenem (AstraZeneca, India) by MIC with the agar dilution method, and results were interpreted according to the guidelines of CLSI (≤4 μg/mL=sensitive, 8 μg/mL=Intermediate, and ≥16 μg/mL=resistant). Quality control of susceptibility testing was done using ATCC 27853 *Pseudomonas aeruginosa*.

## RESULTS

In total, 265 *Acinetobacter* spp. were isolated from 1,242 culture-positive samples from hospitalized patients and were identified up to species level as *A.* *baumannii* (91%) and *A. Iwoffii* (9%). On performing disc-diffusion for antimicrobial susceptibility, *Acinetobacter* spp. showed more than 80% resistance to third-generation cephalosporins. Among quinolones, 81% of the isolates were resistant to ciprofloxacin, while norfloxacin was inactive in 78% cases of nosocomial urinary tract infection (UTI) caused by *Acinetobacter* spp. Among aminoglycosides, although amikacin was relatively effective, still 74% of *Acinetobacter* spp. showed resistance to it. Cefoperazone/sulbactam combination was effective with an overall resistance of 31% while 98% of *Acinetobacter* spp. were resistant to piperacillin. Among carbapenems, 9.1% of the isolates were resistant to imipenem and 9.8% to meropenem (Table 1).

**Table 1:** Antibiotic resistance pattern of *Acinetobacter* species isolated from different wards, expressed in percentage (%)

Antibiotic	Post-operative and others (n=154)	ICU (n=89)	Burns (n=22)	Overall (n=265)
ß-lactams				
Piperacillin	97.9	97.4	100.0	97.9
Carbenicillin	69.6	71.4	50.0	68.8
Cefotaxime	79.0	83.5	83.3	80.8
Ceftazidime	77.6	83.5	83.3	80.0
Cefoperazone	79.0	87.3	77.8	82.3
Imipenem	07.1	12.3	09.1	09.1
Meropenem	07.7	12.7	11.1	09.8
Aminoglycosides				
Gentamicin	83.9	87.3	94.4	85.8
Tobramycin	82.5	86.0	88.9	84.2
Amikacin	74.8	73.4	77.8	74.6
Netilmicin	78.3	83.5	83.3	80.4
Quinolones				
Ciprofloxacin	79.7	83.5	77.8	80.8
Norfloxacin	78.3	71.4	100.0	78.1
Others				
Cefoperazone + sulbactam	27.8	45.6	27.8	31.2

ICU=Intensive care unit

On performing MIC, 39 isolates of *Acinetobacter* spp., which were resistant to meropenem, showed 6.4% and 8.3% of absolute and intermediate resistance respectively. These 39 isolates were recovered from 34 patients whose clinical data revealed that most of these isolates were from patients admitted to intensive care units (Table 2). On further analysis, it was observed that 44.9% of the isolates were on borderline to the moderate/resistance range (Fig.). Interestingly, all carbapenem-resistant/intermediate strains of *Acinetobacter* spp. were also resistant to 12 other antibiotics tested by the disc-diffusion method.

**Fig.**
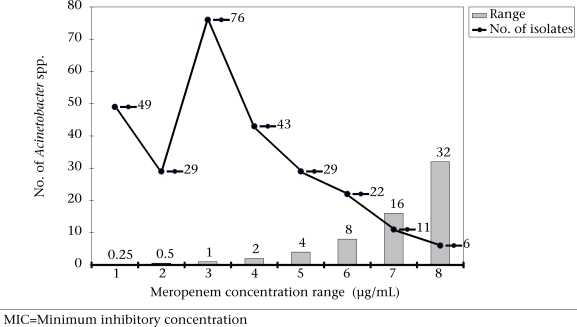
Response of *Acinetobacter* spp. to various concentration ranges of meropenem by MIC

**Table 2:** Clinical data of patients producing carbapenem-resistant Acinetobacters

Sl. no.	Sample	Ward/ICU	Age (years)	Sex	Clinical diagnosis
1	Pus	FSW	45	F	Gall bladder perforation
2	Pus	MSW	34	M	Non-healing ulcer
3	Pus	MSW	33	F	Polytrauma
4	Pus	Spl	50	M	Diabetic foot
5	Blood	NICU	1	M	Neonatal septicaemia
6	Pus	Tr	32	M	Crush injury
7	Pus	Ortho	7	F	Abscess Rt knee
8	Pus	MSW	33	M	Non-healing ulcer
9	Pus	Gyn	28	F	PO infection
10	Swab	ICU	26	M	Multiple fracture
11	ETT	ICU	50	F	Renal failure
12	ETT	ICU	61	M	ARDS, with PUO
13	ETT	ICU	21	F	Respiratory failure
14	Urine	NICU	12 days	M	UTI
15	ETT	ICU	35	M	COPD
16	Pus	MSW	55	M	Necrotizing fascitis
17	Pus	ICU	45	M	Road traffic accident
18	Pus	FSW	35	M	Abdominal surgery
19	Pus	MSW	41	M	Laparotomy
20	Pus	Burns	70	M	90% burn
21	Pus	NICU	1 month	M	Cellulitis
22	ETT	ICU	10	F	Head injury
23	ETT	ICU	35	M	Bronchial asthma
24	Tr. tube	ICU	30	M	Pneumonia
25	Pus	CTVS	45	M	Infective endocarditis
26	Pus	Burns	30	F	70% burn
27	Urine	Gyn	22	F	PO infection
28	ETT	ICU	32	M	COPD, complications
29	Pus	MSW	44	M	Abdominal surgery
30	Tr. tube	ICU	43	F	Bronchial asthma
31	ETT	ICU	70	M	Pneumonia
32	Pus	Spl	48	M	Bracheal artey injury
33	Urine	ICU	29	M	Laparatomy
34	Pus	Surg	27	F	Deglobing injury scalp
35	Cat. tip	ICU	48	F	Opium poisoning
36	ETT	ICU	35	F	GI bleeding, pneumonia
37	Swab	ICU	22	M	Renal failure
38	ETT	ICU	43	F	Pneumonia
39	Blood	ICU	47	M	Septicaemia

ARDS=Acute respiratory distress syndrome; Cat=Catheter; COPD=Chronic obstructive pulmonary disease; CTVS=Cardiovascular thoracic surgery; ETT=Endotracheal tube; FSW=Female surgical ward; GI=Gastrointestinal; Gyn=Gynaecology; ICU=Intensive care unit; MSW=Male surgical ward, NICU=Neonatal ICU, PO=Postoperative; Ortho=Orthopaedics; PUO =Pyrexia of unknown origin; Spl =Special ward; Tr=Tracheostomy; UTI=Urinary tract infection

## DISCUSSION

In the present study, an overall 18% isolation of *Acinetobacter* species in nosocomial colonization/infections was observed. *Acinetobacter* species accounted for 1.4% of all nosocomial infections during 1971–1981 in a university hospital in the United States (8). A more recent study in a university hospital found that hospitalization in an intensive care unit and previous administration of antibiotics were associated with *Acinetobacter* colonization at various sites of the body in 3.2–10.8 per 1,000 patients (9). Contrary to the previous studies, a higher prevalence of *Acinetobacter* spp. in the region could be due to lack of good infection-control practices, personal hygiene, over-crowding situations in infirmary, and heavy patient load.

In this study, more than 75% of the isolates were resistant to third-generation cephalosporins, aminoglycosides, and quinolones. Other studies on Acinetobacters have depicted similar results with respect to these antibiotics (10-13). Thirty-one percent of these isolates was resistant to cefoperazone-sulbactam; the efficacy of this drug was significant (p<0.001) compared to other groups of antimicrobials. However, another study showed 46% resistance to cefoperazone-sulbactam by the disc-diffusion method (11).

Resistance to meropenem was observed in 9.8% of *Acinetobacter* spp. by the disc-diffusion met-hod while 6.4% and 8.3% of the isolates were resistant and intermediate respectively by MIC. Till date, there are limited reports from India on resistance to carbapenem, confirmed by MIC, in the nosocomial isolates of *Acinetobacter* species (5,14). Taneja *et al.* reported a high incidence (>20%) of resistance to carbapenem among Acinetobacters in India. However, a report from France showed that 17% of *Acinetobacter* spp. was resistant to meropenem by the agar dilution method while a study in the UK reported 10% resistance which is quite similar to our results (15,16).

Other studies have shown a high incidence of resistance to carbapenem among Acinetobacters from patients in intensive care units, suggesting that intensive care units are the epicentre for carbapenem-resistant Acinetobacters (17,18).

Meropenem-resistant *Acinetobacter* spp. was also found to be resistant to all other antimicrobials (Pandrug-resistant *Acinetobacter baumannii*) (19). This disturbing situation could be attributed to the increased use of antibiotics which has to be controlled by a strict policy for use of antibiotics, in the face of aggressive marketing by the pharmaceuticals. Effective strategies, such as strict infection-control measures, judicious prescriptions of antibiotics, antimicrobial resistance surveillance programmes, and antibiotic cycling have all been tried successfully to control drug resistance in some countries (20).

Carbapenems have become the drugs of choice in *Acinetobacter-*associated infections in many centres but are slowly being compromised by the emergence of carbapenem-hydrolyzing-lactamases of molecular class B and D (19). Class B carbapenemases found so far in *Acinetobacter*s include various IMP and VIM types; class D enzymes include members of the OXA-23- and OXA-24-related families and various unsequenced types (20). Loss of porins, PBP with reduced affinity, efflux pump, AmpC, and different class B and D ß-lactamases have been associated with resistance to carbapenems in clinical strains of *Acinetobacter* spp. (21,22). A report from India on mechanisms of carbapenem resistance (phenotypic method) among Acinetobacters has suggested that AmpC is responsible for such resistance (5).

Despite the low prevalence of carbapenem resistance in this study, caution has to be exercised in its use in critically-ill hospitalized patients to check any further increase in the resistance to carbapenems. It is notable that almost 45% isolates of *Acinetobacter* species were on the borderline to moderate/resistant range to carbapenem. Regular monitoring and documentation of carbapenem resistance is, therefore, crucial while developing strategies to control infections due to *Acinetobacter* spp. in hospitalized patients.
